# Case Report: a novel chromosomal insertion, 46, XY, inv ins(18;2)(q11.2;q13q22), in a patient with infertility and mild intellectual disability

**DOI:** 10.12688/f1000research.18455.1

**Published:** 2019-03-12

**Authors:** Murat Kaya, İlknur Suer, Şükrü Öztürk, Kıvanç ÇEFLE, Birsen Karaman, Şükrü Palanduz

**Affiliations:** 1 Department of Medical Genetics of Internal Diseases, Istanbul Medical Faculty, İstanbul University, İstanbul, Turkey; 2 Department of Medical Genetics, İstanbul Medical Faculty, Istanbul University, Istanbul, Turkey

**Keywords:** Infertility, mild intellectual disability, insertion

## Abstract

Infertility is an important health problem affecting 15% of couples worldwide. Intellectual disability (ID) is characterized with significant impairment of intellectual function, adaptive daily life skills and social skills. Insertion is a rare chromosomal rearrangement causing infertility and ID. Here, we report a 39-year-old man presenting with primary infertility and mild ID. The patient’s spermiogram was consistent with azoospermia. Conventional cytogenetic analysis showed a novel inversion/insertion type of chromosomal aberration involving chromosomes 18 and 2: 46, XY, inv ins(18;2)(q11.2;q13q22). We carried out the array comparative genomic hybridization analysis to confirm the cytogenetic findings. Y micro-deletion analysis demonstrated that the AZF region as intact. We suggest that the novel insertion found in this case [46, XY, inv ins(18;2)(q11.2;q13q22)] may have caused infertility and mild ID in our patient. To the best of our knowledge, this chromosomal insertion has not previously been reported.

## Introduction

Several factors have been implicated in the pathogenesis of infertility, which are associated with both men and women. Infertility may also occur due to a combined etiology where both male and female factors are combined. In approximately 40% of the cases, the etiology is unclear ^1^.

Balanced chromosomal rearrangements (BCRs), such as autosomal reciprocal translocations, can be related to infertility. The frequency of BCRs in azoospermic men and oligozoospermic men is 0.6% and 1.7%, respectively ^2^.

Intellectual disability (ID) is characterized by significant impairment of intellectual function, adaptive daily life skills and social skills. ID is separated into five groups: mild, moderate, severe, profound and unable to classify ^3^.

ID occurs most likely due to a genetic etiology including BCRs. ID is thought to affect about 1% of the population, of which 85% have mild ID. People with mild ID are slower nearly in all areas of intellectual development, social and daily life skills. These individuals can take care of themselves, learn basic skills associated to safety and health and they may acquire practical life skills ^4^.

In this study, we define an azoospermic male with mild ID who was found to have a novel insertional chromosomal abnormality on conventional cytogenetic and molecular cytogenetic techniques.

## Case Report

A 39-year-old man was referred to our clinic due to infertility. His height and weight were 175 cm and 82 kg, respectively. The patient left school when he was in the third grade of primary school because of learning issues. He was unable to read and write properly, and had deficits in intellectual ability like reasoning or problem solving. Currently, the patient was working as a cleaner in a factory. He was noted to have mild ID.

**Figure 1.**
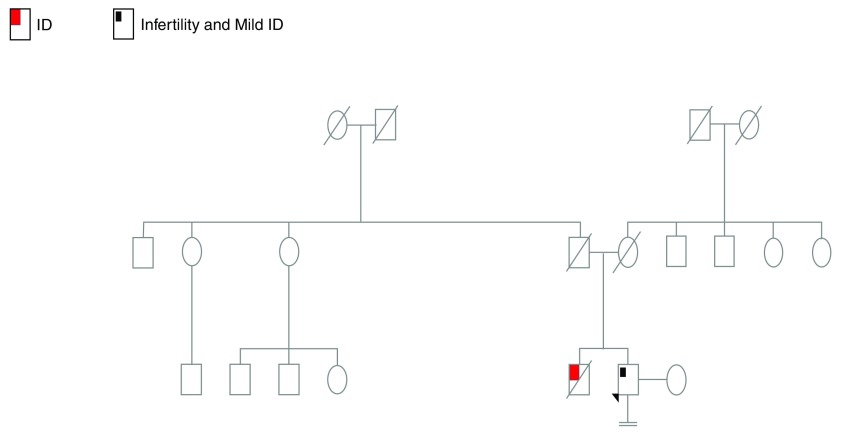
Patient’s pedigree.

On physical examination, the patient had no dysmorphic features. He was married for 8 years; he and his wife were not consanguineous. His parents had two children and the family history of the patient was remarkable for a deceased brother at the age of 15 years, who had also ID ( Figure 1). Since there was no history of spontaneous pregnancy during his marriage, the patient was considered to represent a case of primary infertility who had also mild ID. Sperm analysis showed complete azoospermia. *In vitro* fertilization was performed four times by testicular sperm extraction without success. Luteinizing hormone, follicular stimulating hormone and testosterone levels were compatible with hypergonadotropic hypogonadism ( Table 1). Y micro-deletion analysis demonstrated that AZFa, AZFb and AZFc regions on the Y chromosome were intact. After conventional cytogenetic analysis, we performed array conventional cytogenetic technique (aCGH). Karyotype analysis could not be performed for the parents or the patient’s brother, since they were not alive.

**Table 1.** Hormone levels of the patient.

Hormone	Patient	Reference range
Luteinizing hormone (IU/L)	14.87 ↑	1.7–8.6
Testosterone (ng/dL)	1.89 ↓	2.18–9.06
Follicular stimulating hormone (mIU/ml)	24.25 ↑	1.5–12.4

*Conventional cytogenetic technique:* Peripheral blood lymphocytes were used for a 72-hour culture. Chromosome analysis was performed on phyto-haemagglutinin-induced peripheral blood lymphocytes. Metaphase plaques were analyzed using the GTG banding method at almost 500–550 band resolution.

*aCGH:* An Agilent SurePrint G3 CGH+SNP Microarray Kit (4x180K) was used for genetic analysis of the patient. Microarray data were analyzed using Feature Extraction and Agilent Cytogenomics v4.0.3.12 software. Log ratios between -0.5 – 0.5 and variations with less than 5 consecutive probes were excluded. Genomic positions were based on GRCh37/hg19 *Homo sapiens* assembly.

**Figure 2.**
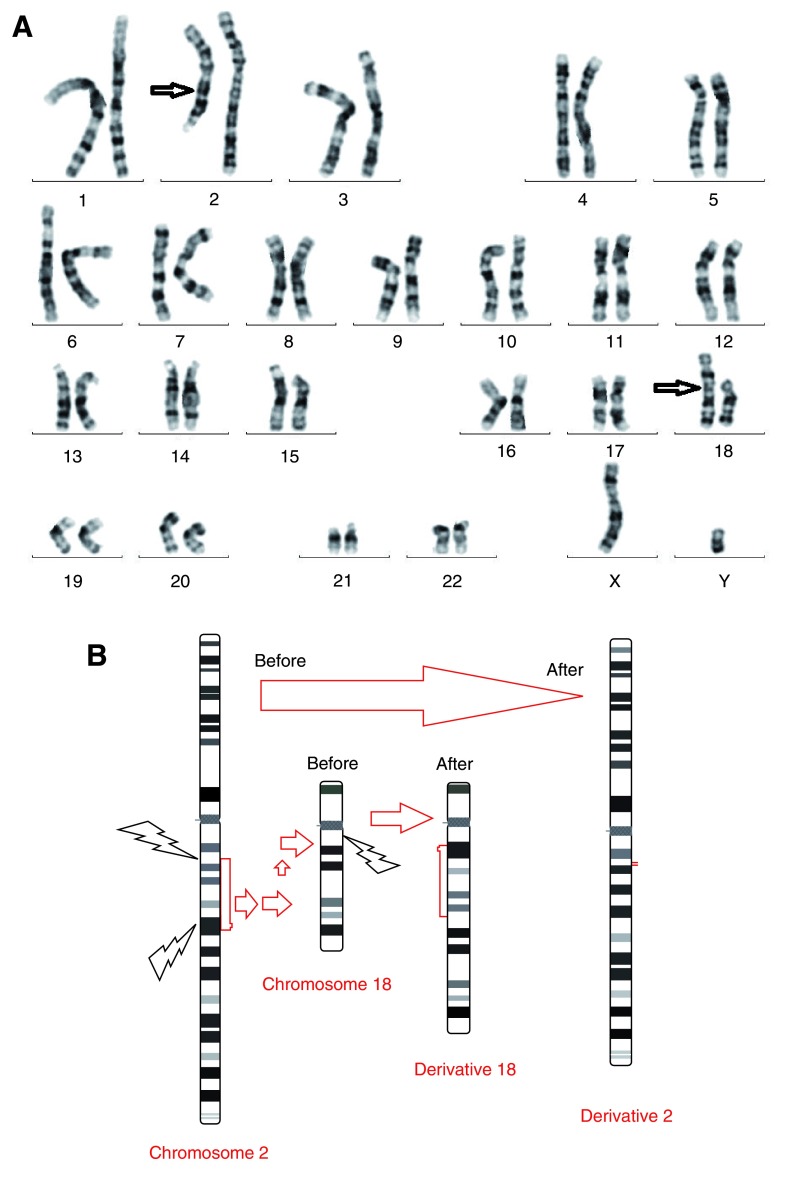
Image of the patient's chromosomes. **a)** Patient’s GTG banded karyotype, **b)** Schematic of normal and derivative chromosomes 2 and 18 and breakpoints regions of chromosomes.

**Figure 3.**
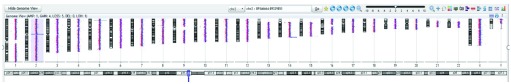
Array-based comparative genomic hybridization chromosomal ideogram and clustering of breakpoints.

Conventional cytogenetic analysis revealed that the patient had an insertional translocation: 46, XY, inv ins(18;2)(q11.2;q13q22) ( Figure 2). Array CGH did not show any deletion or duplication ( Figure 3).

## Discussion

Insertions occur after at least three breaks on chromosomes ^5^. Incidence of insertional chromosome translocation is nearly 1:80.000 live births ^6^. Molecular mechanisms of chromosomal insertions are not well understood ^5^. An individual with an inter-chromosomal insertion can transmit unblanced chromosomes to his/her child with a theoretical risk of 50% in each pregnancy ^6^.

Insertional translocations affecting gene function may cause abnormal phenotype in different ways. According to one hypothesis, the derivative chromosomes formed after insertional translocation can contain gene or genes with increased expression. A second hypothesis states that structure of a gene located at the breakpoint may be disturbed and as a result either loss or gain of function can occur. Lastly, genes located at the breaking segment can be deleted or duplicated which can give rise to gene expression abnormalities owing to position effects ^6^. In addition to these genetic mechanisms, insertional translocations can also affect the genome by epigenetic means. For instance, some microRNAs may be located in the break regions. It is thought that nearly 60% of all human genes are regulated by microRNAs. Furthermore, many microRNAs are located in fragile regions of the genome and considered to have an important role in the pathogenesis of many diseases ^7^.

Classical chromosome analysis revealed that our patient carried an insertional translocation, 46, XY, inv ins(18;2)(q11.2;q13q22). To the best of our knowledge, this chromosomal insertion has not previously been reported. aCGH is used to detect genomic micro-deletions/duplications (copy number changes). However, aCGH failed to detect any micro-deletion or micro-duplication at the breakpoint regions of this insertional translocation [arr(1-22)×2,(XY)×1]. Although aCGH is a useful and a modern technique, it has some limitations. Mosaicism cannot be detected and BCR may be missed by this method. Furthermore, small deletions or duplications which are less than 10 kb can be difficult to detect with aCGH. Although conventional cytogenetic analysis is an old technique, it is still the cheapest and most useful method to obtain a general information about whole chromosomes rapidly ^8^.

Li and colleagues elucidated the insertional translocation 46,XY inv ins(18,7) (q22.1; q36.2q21.11) found in an azoospermic man with next generation sequencing (NGS). It was demonstrated that two disrupted genes, DPP6 and CACNA2D1, were at breakpoint sites of the chromosomes, suggesting they may be associated with azoospermia. Moreover, neither micro-deletions nor duplications were detected at these breakpoint regions ^9^.

BCRs detected by conventional karyotype analysis can be found to be unbalanced at the molecular level. We suggest that these chromosomal regions affected in our patient can be evaluated with advanced molecular techniques like NGS. Such an approach could unveil sequence abnormalities, which would potentially shed light on the molecular pathogenesis of azoospermia and/or ID.

## Consent

Informed written consent for the publication of clinical details and images was obtained from the patient.

## Data availability

All data underlying the results are available as part of the article and no additional source data are required.
