# Supplementary material for: Case Report: a novel chromosomal insertion, 46, XY, inv ins(18;2)(q11.2;q13q22), in a patient with infertility and mild intellectual disability
Source: F1000Res. 2019 Mar 12;8:281. [Version 1] doi: 10.12688/f1000research.18455.1 (PMC6567292; doi:10.12688/f1000research.18455.1)
Supplement: Peer review history

# Peer review: Reviewer response for version 1

**DOI:** 10.5256/f1000research.20192.r48939

**Published:** 2019-06-13

**Authors:** Ender Karaca

**Affiliations:** 1 Department of Genetics, University of Alabama at Birmingham, Birmingham, AL, USA

Murat Kaya *et al.* report a male patient with mild intellectual disability and infertility. The authors used conventional G-banding, FISH and aCGH methods in order to investigate genetic etiology in the patient. They also provide the levels of luteinizing hormone, follicular stimulating hormone and testosterone, which were compatible with hypergonadotropic hypogonadism. Through their investigation they were able to detect an apparently balanced inverted inversion, 46,XY,ins(18;2)(q11.2;q22q13). A follow up aCGH analysis (using the platform Agilent aCGH+SNP Microarray, 4x180K) could not detect any unbalances at the break points involved in the insertion event. They also excluded Y micro-deletion via analyzing AZFa, AZFb and AZFc regions on the Y chromosome.

 Overall the case report is worth being indexed and clinicians and researchers who work on infertility and ID will benefit from it. However, it needs to be revised in order to correct some points and to improve the value of the presented clinical and genomic data. I explained my specific concerns below.

**Title:**

1. Please revise the cytogenetic nomenclature based on ISCN 2016. There should be no space after commas and you don’t need to use “inv”, you actually just have to change the order of the break points in chromosome 2 in the nomenclature according to the orientation of the inserted fragment. So the nomenclature will be: “46,XY,ins(18;2)(q11.2;q22q13)”.

 

**Abstract:**

1. The abstract is well written, however the first 3 sentences could benefit from revising which would provide a better fluency. This revision could include the removal of the definition of ID, the second sentence of the abstract, as this definition is repeated a couple of times in the introduction. I’d sincerely recommend an entrance to the abstract:
  “ *Infertility and intellectual disability (ID) are important health problems affecting 15% of couples worldwide and 1-2% of the general population, respectively. Chromosomal aberrations including insertions are responsible for both infertility and ID in many cases." *
  Of course, this is just a minor issue and the authors are free to consider or not.
2. The authors say in the abstract “We carried out the array comparative genomic hybridization analysis to confirm the cytogenetic findings”. How do they expect to confirm a balanced rearrangement with aCGH? Actually, in the discussion they say “aCGH is used to detect genomic micro-deletions/duplications (copy number changes). However, aCGH failed to detect any micro-deletion or micro-duplication at the breakpoint regions of this insertional translocation [arr(1-22)×2,(XY)×1].”, and this sounds like the aCGH method has been used to detect any potential copy number change at the break points, which is indeed a more appropriate reason to use aCGH method in this case. So, please revise the statement in the abstract according to your statement in the discussion.   

 

**Introduction:**

 The introduction would benefit from giving a little more detail from the background of both intellectual disability and infertility. I would rather revise the introduction in a way which will address the following questions:

 (Paragraph for infertility)

1. What is the definition of infertility?
2. What is the frequency of infertility in the general population (preferably providing male and female infertility ratios)?
3. What type of genetic causes can lead to infertility (i.e. molecular, cytogenetic)?
4. What is the frequency of BCRs in the etiology of infertility?

 

 (Paragraph for ID)

1. What is the definition of ID?
2. What is the frequency of ID in the general population?
3. Do all IDs result from genetic causes?
4. What type of genetic causes can lead to IDs (i.e. molecular, cytogenetic)?
5. What is the frequency of BCRs in the etiology of ID?

(Paragraph that provide literature summary for cases with both infertility and ID)

1. Are there any cases in the literature representing with both infertility and ID? If so, what type of genetic cause(s) are reported in these patients? Is there any example for such a case (with both ID and infertility) who has BCRs?  

The last paragraph will summarize your findings as in the current version. However, the authors need to correct the statement: "In this study, we define an azoospermic male with mild ID who was found to have a novel insertional chromosomal abnormality on conventional cytogenetic and molecular cytogenetic techniques." as it means that they could detect the chromosomal rearrangement on aCGH too, which is not true.  

**Case Report:**

1. ID in the patient was classified as mild. Is there an IQ test score?
2. Is there any medical record of an IQ test for the affected brother? Did he have mild ID too?
3. Did his wife undergo any investigation for infertility? Please provide the clinical data - if available - whether the female infertility could be excluded or not.
4. It would be more informative for readers (especially for clinical genetics trainees) to provide images of the Y-microdeletion analysis, even in the extended data section.
5. Please correct "array conventional cytogenetic technique" to "array comparative genomic hybridization", which correctly corresponds to abbreviation "aCGH".
6. Please change "phyto-haemagglutinin-induced" to "phytohaemagglutinin-induced". You do not need to use dash within the word "phytohaemagglutinin".​​​​​​

 

**Discussion:**

1. In the second paragraph of the discussion, the authors mention about two hypotheses regarding the potential mechanisms by which insertions cause abnormality. Can they provide reference(s) for these two hypotheses?
2. The statement “For instance, some micro-RNAs may be located in the break regions” may cause confusion especially for clinicians who do not know the details of microRNA functions and the mechanisms that they use in the regulation of gene expression. The current sentence could mean either “micro-RNA coding genes” or “the genomic region that is a target for micro-RNA”, please clarify.
3. Could the authors provide reference for the statement “It is thought that nearly 60% of all human genes are regulated by microRNAs”?
4. In the 3 ^rd^ paragraph of the discussion, the authors claim that mosaicism cannot be detected by aCGH. Although aCGH has significant disadvantages with detecting low-level mosaicism, mosaicisms with the percentage over 20-25 (>20-25%) can be detected by most of the recent aCGH platforms. Thus, the statement "mosaicism cannot be detected" is misleading; please correct it.
5. The authors cite a report in which an inverted insertion “46,XY inv ins(18,7) (q22.1; q36.2q21.11)”. This nomenclature is wrong as well. Please consider ISCN 2016 as a reference for cytogenetic nomenclature. In this cited report, both *DPP6* and *CACNA2D1* are located on chromosome 7, while the overlapping chromosome that is involved in the rearrangement in both reports is chromosome 18. It would be interesting to know if there is any known or candidate gene for infertility and/or intellectual disability located in any of the 3 break points involved; 18q11.2, 2q13, or 2q22.
6. It seems that the most likely reason for infertility and ID in the reported male is the chromosomal rearrangement. However, it could be also possible that the given insertional translocation does not cause any of the ID or infertility in this patient. There may be a different mutation in a known gene which could be detected by sequencing methods. Even more, this insertion could be associated with just one of the phenotypes; infertility or ID; while a second mutation in another locus causes the other phenotype. So, NGS (WES or WGS) analysis could be helpful to further evaluate the break points as mentioned by the authors, moreover NGS could also identify a different locus that is not involved the current rearrangement, which could contribute to the phenotype of the patient. Please also discuss these etiologic possibilities in the discussion.

**Figure 1:**

1. The pedigree looks like compressed from both sides. Please consider keeping the original square and circle shapes while you try to fit the image in the page.

**Figure 2:**

1. I want to thank to authors for this terrific G-banding.
2. This is also a very nice demo. It would be better to designate the bands where break points are located (2q13, 2q22, and 18q11.2). The red arrows are not necessary, since you labeled each chromosome (chromosome 2 vs derivative 2, etc.).

**Figure 3: **

1. Rather than showing only entire chromosome plotting with very limited detail, you can also provide individual array plots of chromosome 2 and 18, which will be easy to evaluate.  

I have read this submission. I believe that I have an appropriate level of expertise to confirm that it is of an acceptable scientific standard.

# Peer review: Reviewer response for version 1

**DOI:** 10.5256/f1000research.20192.r46870

**Published:** 2019-04-17

**Authors:** Murat Erdogan

**Affiliations:** 1 Department of Medical Genetics, Kayseri Training and Research Hospital, Kayseri, Turkey; 2 Turkish Society of Medical Genetics, Ankara, Turkey

Infertility and ID are important health problems. There are several reasons in the etiology of each of the two diseases, but genetic causes are more important. Among the genetic reasons, chromosomal rearrangements are remarkable. In this case, both infertility and mild-ID are seen. Genetic studies in this case indicate a chromosomal rearrangement that has not previously been in the literature. In the diagnostic pathway, physical examination and family history of the patient were first investigated. No dysmorphic features were observed in the patient. Then, routine laboratory tests were performed, and azoospermia was detected in the patient. In addition, according to the information obtained from patient, in vitro fertilization process failure was noted. Hypergonadotropic hypogonadism was also observed in the patient. According to the algorithm, genetic tests to be performed for the diagnosis of both infertility and ID were started with conventional cytogenetics and continued with aCGH. As a result, a balanced chromosomal translocation was detected in the patient. Insertional translocations affect various genetic mechanisms and disrupt gene functions. This type of translocation may lead to increased or decreased gene functions. These chromosomal translocations may also affect epigenetic mechanisms. If the affected genes in chromosomal fracture regions can be determined with more advanced methods, they will contribute to the etiology of infertility and ID.

 Some minor corrections may be required in this case. It is necessary to determine the number of areas studied during cytogenetic analysis, to indicate whether the change is in all areas, and the pedigree drawing shown in Figure 1 should be redrawn and numbered according to the basis principles. In order to clearly understand the fracture regions shown in Figure 2, it is useful to show to centromere subregions in the ideogram.

I have read this submission. I believe that I have an appropriate level of expertise to confirm that it is of an acceptable scientific standard.

## References

[1] CeylanGGCeylanCElyasH: Genetic anomalies in patients with severe oligozoospermia and azoospermia in eastern Turkey: a prospective study. *Genet Mol Res*.2009;8(3) : 10.4238/vol8-3gmr616 915-22 10.4238/vol8-3gmr616 19731213
